# Genetic test results and one-year developmental outcomes of fetuses with congenital heart disease

**DOI:** 10.3389/fped.2025.1518784

**Published:** 2025-03-28

**Authors:** Hui Hu, Bo Zhou, Qunda Shan, Shuangshuang Shen, Xiangdong Zhang, Penglong Chen, Jiao Liu, Xiaofang Lan

**Affiliations:** ^1^ Prenatal Diagnostic Center, Lishui Maternity and Child Health Care Hospital, Lishui, Zhejiang, China; ^2^ Department of Cardiology, Bishan Hospital of Chongqing Medical University, Chongqing, China; ^3^ Prenatal Diagnostic Center, Jinhua Maternity and Child Health Care Hospital, Jinhua, Zhejiang, China

**Keywords:** congenital heart disease, SNP array, chromosome, prenatal diagnosis, short-term outcomes

## Abstract

**Objective:**

This study evaluated the utility of single nucleotide polymorphism (SNP) microarray analysis in prenatal genetic assessment of fetuses diagnosed with congenital heart disease (CHD), retrospectively analyzing pregnancy outcomes and their association with physical and intellectual development within the first year of life.

**Patients and methods:**

It included 105 fetuses diagnosed with CHD via prenatal echocardiography from January 2016 to June 2020, categorized into two groups: isolated cardiac structural abnormalities (76 cases) and additional extracardiac structural abnormalities (29 cases). All fetuses underwent chromosome karyotype and SNP array testing, with retrospective analysis of pregnancy outcomes, postnatal physical and intellectual development at one year of age.

**Results:**

Chromosomal abnormalities were identified in 15.2% (16/105) of the fetuses. A significantly higher incidence of chromosomal abnormalities was observed in the group with combined extra-cardiac structural abnormalities compared to the group with isolated cardiac abnormalities (*P* < 0.05). The detection rates of pathogenic Copy Number Variations (CNV), variants of uncertain significance (VOUS), and benign CNV showed no significant differences between the groups (*P* > 0.05). The detection rate of CNV was significantly lower in fetuses with isolated cardiac abnormalities (*P* < 0.05). The delivery rate was 61.0%, significantly higher in fetuses with only cardiac abnormalities. Of these, 38.5% of ventricular septal defects closed naturally within the first year. Only a small proportion of the children displayed developmental delays at one year of age.

**Conclusion:**

SNP array analysis enhances the detection of genetic etiologies in CHD, assisting in the precise localization of chromosomal anomalies and candidate gene screening. It is effective for prenatal diagnosis in CHD fetuses. Fetuses with isolated cardiac structural abnormalities show lower rates of chromosomal anomalies and CNVs and generally have favorable one-year developmental outcomes, underlining the importance of SNP array analysis in managing CHD outcomes.

## 1 Introduction

Congenital heart disease (CHD), comprising a wide variety of anomalies and malformations involving the heart and great vessels that develop *in utero* during the formation of the cardiovascular system, manifests as structural and functional abnormalities present at birth (1, 2). The prevalence of congenital heart disease varies between 8 and 10 per 1,000 newborns globally, depending on diagnostic methods and definitions used (3, 4). Recent estimations indicate that approximately 0.5 million adults in the United States and 12 million adults globally have emerged as survivors of CHD, By 2017, CHD had risen to become one of the top eight causes of infant mortality across all Socio-demographic Index quintiles (5), in-addition, The prevalence of CHD has shown an increasing trend, rising from 0.6 per 1,000 live births in 1930 to 9.1 per 1,000 live births after 1995 (4, 6). Due to significant advancements that have been made in cardiovascular diagnostics and cardiothoracic surgery over the past century, resulting in substantial improvements in the success rate of surgery and long-term postsurgical survival rates for severe forms of CHD (7). As a result, an entirely new and continuously expanding patient population has emerged: individuals with grown-up congenital heart disease (GUCH), Up to 52% of patients with CHD develop post-traumatic stress disorder (8), GUCH patients frequently require long-term specialized medical care, leading to high healthcare-related costs. Consequently, the global health burden attributable to CHD is rapidly escalating (6). Hence, there is a pressing need to prioritize the search for the underlying causes of CHD and the identification of associated inherited syndromes in current prenatal diagnosis practices for CHD (9).

Currently, the exact pathogenesis of CHD remains unclear; it is generally believed to be associated with exposure to teratogenic agents in the environment or genetic factors (10, 11), Research conducted on families has shown robust genetic predispositions contributing to the development of CHD (11), Genetic factors play a prominent role in the pathogenesis of CHD among various etiological factors, encompassing chromosomal abnormalities, gene mutations, and copy number variations (CNV) (11, 12). Prenatal detection of CHD through genetic testing has revolutionized the management and prognostication of affected fetuses. Understanding the genetic underpinnings of CHD is crucial for accurate diagnosis, counseling, and potential intervention strategies.

Currently, The four techniques for assessing CHD genetic information are chromosome karyotype, Molecular DNA Testing, Fluorescence *in situ* Hybridization (FISH), and Chromosomal Microarray Analysis (CMA) (13). while these techniques can detect larger chromosomal abnormalities and CNVs, they might miss smaller variants or rare mutations. Traditional chromosome karyotype analysis can only detect abnormalities in chromosome number or large segments of chromosome structural variations (14). FISH, including its restricted diversity and inability to provide comprehensive genome-wide information, constrain its applicability (15). In recent years, advancements in genetic testing techniques, particularly CMA and single nucleotide polymorphism (SNP) arrays, have enabled comprehensive evaluation of chromosomal abnormalities and CNV associated with CHD.

CMA is a recently developed molecular technique that allows for the identification of small segments of chromosome deletions or duplications, as well as the determination of the location of the variation. Similarly, SNP arrays recognize chromosomal microdeletions or microduplications and pinpoint the location of the variation (16). These technologies have provided unprecedented insights into the genetic landscape of CHD, shedding light on both common and rare genetic anomalies contributing to its pathogenesis (17). In recent years, numerous scholars have conducted research on the application of CMA in CHD, confirming the association between CHD and chromosomal microdeletions and/or microduplications (18–21), However, the precise role of SNP array analysis in prenatal genetic diagnosis and its utility in clinical decision-making for CHD fetuses warrants further clarification. The aim of this study was to investigate the genetic profile of fetuses with CHD, focusing on chromosomal abnormalities and CNV detected through SNP array analysis, and to evaluate the association between genetic anomalies, pregnancy outcomes, and postnatal development.

## 2 Patients and methods

### 2.1 Patients

This study selected 105 fetuses diagnosed with CHD through echocardiography examinations at prenatal diagnostic centers between January 2016 and June 2020. Among them, 67 cases were from the Maternal and Child Health Hospital of Lishui, and 38 cases were from the Maternal and Child Health Hospital of Jinhua. Inclusion criteria were as follows: (1) Prenatal ultrasound diagnosis indicated CHD in the fetus; (2) Pregnant women and their families provided informed consent to participate in the study. Exclusion criteria included: (1) Pregnant women with a family history of genetic diseases; (2) Pregnant women with severe pregnancy-related diseases or exposure to adverse environments: This included conditions such as preeclampsia, gestational diabetes mellitus, and other pregnancy-induced hypertensive disorders that could independently affect fetal outcomes or complicate follow-up. (3) Incomplete clinical data; (4) Low compliance, making follow-up and data collection difficult. The types of CHD included 19 cases of conotruncal anomalies, 53 cases of septal defects, 5 cases of left ventricular outflow tract obstruction (LVOTO), 4 cases of right ventricular outflow tract obstruction (RVOTO), and 24 cases of other cardiac malformations. LVOTO was characterized by restricted flow at the aortic valve or aortic arch, while RVOTO was characterized by echocardiographic findings of restricted flow through the pulmonary valve or artery. Depending on whether the fetus had extracardiac structural abnormalities, they were divided into a group with isolated cardiac structural abnormalities (76 cases) and a group with associated extracardiac structural abnormalities (29 cases). Extracardiac abnormalities included single umbilical artery, choroid plexus cysts, femoral shortening, wide interpupillary distance, etc. Both groups of pregnant women had no history of pregnancy-related infections, severe pregnancy-induced hypertension, or diabetes. There were no statistical differences in the age of pregnant women or gestational weeks of fetuses between the two groups. This study was approved by the ethics committees of the two hospitals mentioned above, and informed consent was obtained from pregnant women.

### 2.2 Methods

All fetuses underwent chromosomal karyotype analysis. Fetuses with normal karyotype analysis and those with suggested structural abnormalities underwent SNP array testing. Fetal samples were obtained via amniocentesis or umbilical cord blood, and peripheral blood samples were collected from the parents. Pathogenic CNV were analyzed, and candidate pathogenic genes within the identified segments were determined. Follow-up assessments were conducted on pregnancy outcomes and fetal development, including whether extracardiac structural abnormalities were present.

#### 2.2.1 Chromosomal karyotype analysis

Under sterile conditions, 20 ml of amniotic fluid was extracted, and conventional amniotic fluid cell culture, harvesting, and slide preparation were performed using the trypsin digestion method, followed by G-banding. For pregnancies of 26 weeks or more, 1–2 ml of umbilical cord blood samples were cultured, harvested, prepared on slides, and subjected to G-banding. Twenty cells were counted under a microscope, with doubling in case of suspected sex chromosome abnormalities or addition up to 100 cells in case of suspected mosaicism, and 5 karyotypes were analyzed.

#### 2.2.2 SNP array testing

① Genomic DNA extraction: Genomic DNA from amniotic fluid, umbilical cord blood, or peripheral blood was extracted using the Qiagen DNA kit from Germany. ② SNP testing: The standard operation procedure of genomic DNA digestion, ligation, amplification, purification, fragmentation, labeling, hybridization, washing, and scanning were performed using the Affymetrix SNP array detection platform and Cyto Scan 750 K chip. Data analysis was conducted using the ChAS software. ③ Determination of test results: The detected CNV were compared and analyzed against public databases such as DGV, OMIM, UCSC, and DECIPHER. CNV were categorized based on their detected nature into pathogenic CNV, likely pathogenic CNV, CNV with clinically unclear significance, likely benign CNV, and benign CNV. CNV categorized as likely pathogenic, clinically unclear significance, and likely benign were classified as VOUS (Variants of Unknown Significance). Peripheral blood samples from the parents of fetuses with VOUS were further subjected to SNP array testing to determine the origin of fetal CNV and assist in assessing the nature of the variants. Families were informed about the clinical implications of VOUS findings and were advised to seek genetic counseling to better understand the potential impact on short-term outcomes.

#### 2.2.3 Follow-up

All fetal pregnancy outcomes were observed and recorded. After delivery, information such as gestational age and birth weight of the newborns were documented. At one year of age, assessments of head circumference, weight, and height were conducted according to the WHO Standards (2006 Edition). Assessment indicators included head circumference/age, weight/age, and height/age. Median (M) values plus or minus standard deviations (SD) were used to evaluate physical growth, with classifications into lower (<M − 2SD), normal (M ± 2SD), and higher (>M + 2SD) categories. If any parameter fell below the lower category, it was classified as delayed physical development. Intellectual development was assessed using the Neuropsychological Development Scale for Children (0–6 years, 2008 Edition). Assessment included tests for gross motor skills, fine motor skills, adaptive abilities, language skills, and personal-social behavior. Results were represented using developmental quotient (DQ), with DQ ≥ 85 indicating normal intelligence, 85 > DQ ≥ 70 indicating low intelligence, and DQ < 70 indicating intellectual disability.

#### 2.2.4 Statistical methods

Data analysis was performed using SPSS 22.0 statistical software. Multivariable logistic regression models were employed to account for potential confounders, including maternal age, gestational age, and the presence of extracardiac abnormalities. Between-group differences were analyzed using the *χ*^2^ test (with continuity correction *χ*^2^ test or Fisher's exact test), and *P* < 0.05 was considered statistically significant.

## 3 Result

### 3.1 Overall distribution of genetic testing results in fetuses with congenital heart disease

**Figure 1:**
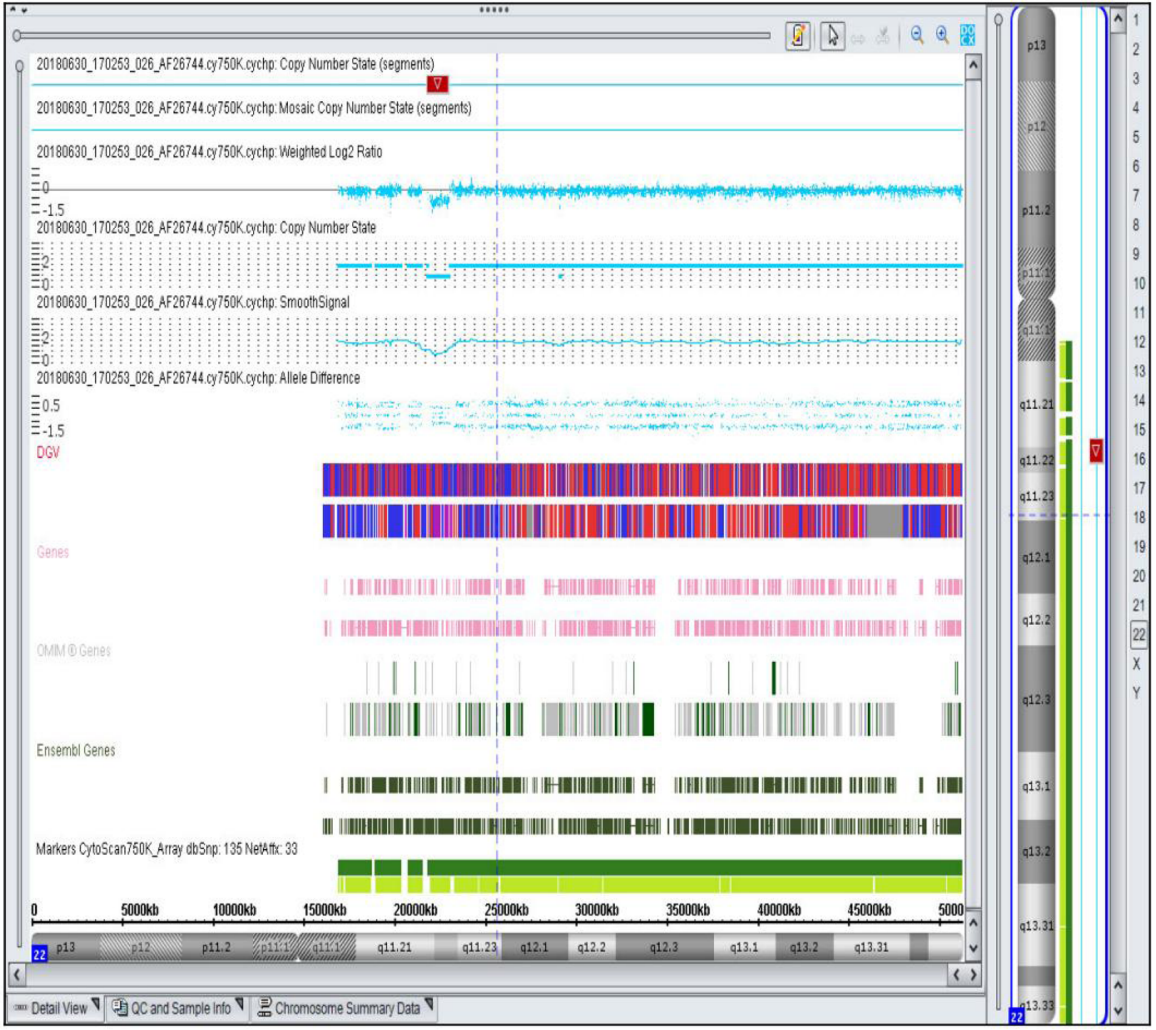
Representative SNP array result showing a 1.1 Mb microdeletion in the 22q11.21q11.22 region of chromosome 22 detected in a fetus with ventricular septal defect. The deletion includes 10 OMIM genes, such as MAPK1 (176948). The highlighted region indicates the specific deletion.

Among the 105 fetuses, chromosomal G-banding karyotype analysis revealed chromosomal abnormalities in 16 cases, with a detection rate of 15.2% (16/105). The detection rate of chromosomal abnormalities in the group with associated extracardiac structural abnormalities (31.0%) was significantly higher than that in the group with isolated cardiac structural abnormalities (9.2%) (*P* < 0.05). There were no statistically significant differences among the detection rates of pathogenic CNV, VOUS, and benign CNV between the two groups (*P* > 0.05). The probability of not detecting CNV in the group with isolated cardiac structural abnormalities (69.7%) was significantly lower than that in the group with associated extracardiac abnormalities (44.8%) (*P* < 0.05) (Table 1, Figure 1).

**Table 1:** Distribution of chromosomal karyotype abnormalities and CNV in 105 cases of congenital heart disease fetuses.

CHD type	Count	Chromosomal abnormalities	Chromosomal microarray analysis (normal karyotype)			
			Pathogenic CNV	VOUS	Benign CNV	NO CNV
Isolated cardiac malformation	76	7 (9.2)	5 (6.6)	7 (9.2)	4 (5.3)	53 (69.7)
Extracardiac cardiac abnormalities	29	9 (31.0)	4 (13.8)	2 (6.9)	1 (3.4)	13 (44.8)
*χ^2^*		6.14	0.63	0.00	—	5.58
*P*		0.01	0.43	0.99	1.00	0.02

CNV, copy number variations; VOUS, variants of uncertain significance.

### 3.2 Chromosomal karyotype analysis results in CHD fetuses

Chromosomal G-banding karyotype analysis revealed chromosomal abnormalities in 16 out of 105 fetuses, with a detection rate of 15.2% (16/105). These abnormalities included: 13 cases of numerical abnormalities (6 cases of trisomy 21, 6 cases of trisomy 18, and 1 case of X-trisomy) and 3 cases of structural abnormalities [1 case of deletion in the 10p14 chromosomal region, 1 case of deletion in the 1q chromosomal region, and 1 case of chromosomal translocation t(4;10)(p14;q25)] (Table 2).

**Table 2:** General information and SNP array testing results of 16 fetuses with detected chromosomal abnormalities.

ID	Gestational age	Chromosomal karyotype analysis	SNP array analysis	Ultrasound findings	Extracardiac abnormalities	Pregnancy outcomes
1	19 + 2	46, XN, der(10)t(4;10)(p14;q25)	4p16.3p14 (Duplications 39.7 Mb); 10q26.13q26.3 (Deletion 10.8 Mb)	DORV, PAS, VSD	—	Termination
2	20 + 5	47, XN, +21	Trisomy 21	VSD	—	Termination
3	24 + 3	47, XN, +21	Trisomy 21	SA, TA, RVH, VSD,MR	Short Femur	Termination
4	25	47, XN, +21	Trisomy 21	FPVE,APARI	—	Termination
5	28 + 6	47, XN, +18	18p11.32q23 (Duplications 77.8 Mb)	VSD	SUA	Termination
6	26	47, XXX	Triple X	TVR	SUA	Term, C-section
7	19 + 4	47, XN, +21	Trisomy 21	VSD	—	Termination
8	29	47, XY, +18	Trisomy 18	TVS, RVOTS, PAS, RVH, MRCAF	—	Termination
9	26	47, XY, +18	Trisomy 18	d-TGA, VSD	Bilateral lateral ventricular choroid cysts	Termination
10	23 + 4	47, XY, +18	Trisomy 18	VSD	Choroid cysts, small gastric bubble	Termination
11	23	46, XN, del(10)(p14)	10p15.3p14 (Deletion 10.1 Mb); 16p13.3 (Duplications 1.1 Mb)	VSD, dextrocardia	Bilateral choroid cysts, Wide interocular distance	Termination
12	24	46, XY, del(1)	1q43q44 (Deletion 9.8 Mb); 5q35.3 (Duplications 1.6 Mb)	TOF	SUA	Termination
13	23	47, XY, 1qh+, +21	Trisomy 21	VSD	Bilateral choroid cysts	Termination
14	26	47, XY, +21	Trisomy 21	CAVSD	—	Termination
15	24	47, XX, +18	Trisomy 18	VSD	Bilateral choroid cysts	Termination
16	26	47, XX, +18	Trisomy 18	VSD	—	Term, C-section

APARI, aortic-pulmonary artery ratio imbalance; CAVSD, complete atrioventricular septal defect; DORV, double outlet right ventricle; d-TGA, dextro-Transposition of the great arteries; FPVE, fetal pulmonary valve enhanced; MR, mitral regurgitation; MRCAF, multiple right coronary artery fistulas; PAS, pulmonary artery stenosis; RVH, right ventricular hypoplasia; RVOTS, right ventricular outflow tract stenosis; SA, single atrium, SUA, single umbilical artery, TOF, tetralogy of fallot; TA, tricuspid atresia; TVR, tricuspid valve regurgitation, TVS, tricuspid valve stenosis; VSD, ventricular septal defect.

### 3.3 SNP array testing results in CHD fetuses

Among the 89 CHD fetuses with normal karyotypes, 23 cases (25.8%, 23/89) were found to have CNV in their genomes. Among these, 9 cases (10.1%, 9/89) were identified as pathogenic CNV. The sizes of the pathogenic CNV detected ranged from 1.0 to 13.1 Mb. Among them, 7 cases were known microdeletion/microduplication syndromes, including 22q11.2 distal deletion syndrome (Figure 1), DiGeorge syndrome, Xq27.3-q28 duplication syndrome, 1p36 deletion syndrome, cat-eye syndrome, 16p13.11 microdeletion syndrome, and Miller-Dieker syndrome. Additionally, 2 cases were newly identified, with deletion fragments larger than 10 Mb, containing multiple known pathogenic genes. Database comparison analysis confirmed their pathogenicity (Table 3). Among the fetuses with pathogenic CNV, SNP array testing was conducted for 7 parents, revealing that 2 cases inherited the CNV from their mothers, while 5 cases were *de novo* mutations. Initially, 12 cases had clinically unclear significance CNV, of which 7 cases refused parental SNP array validation. The remaining 5 cases underwent SNP array testing for parents, revealing that 3 fetal CNV originated from phenotypically normal mothers or fathers, thus classified as benign CNV. Therefore, 9 cases were finally classified as VOUS, and 5 cases were classified as benign CNV.

**Table 3:** Clinical data and analysis results of 9 cases of pathogenic CNV fetuses.

ID	Gestational age	CNV location (deletion/duplication)	Fragment length	Genetic origin	DECIPHER database ID or known syndrome	OMIM genes	Cardiac type	Extracardiac abnormalities	Pregnancy outcomes
17	25 + 1	22q11.21q11.22 (Deletion)	1.1 Mb	Mother	22q11.2 distal deletion syndrome	Deletion: MAPK1, SMARCB1	VSD	—	Premature birth, vaginal delivery
18	27	22q11.21 (Deletion)	3.1 Mb	Mother	DiGeorge syndrome	Deletion: TBX1, CRKL, DGCR6	RAA, PLSVC	—	Termination
19	23	9p24.3p24.1 (Duplication)	5.3 Mb	*de novo*	DECIPHER 373428; DECIPHER 307196	Duplication: DOCK8, SMARCA2	CAVSD, PTA	Bilateral ventricular enlargement	Termination
		11q24.1q25 (Deletion)	12.3 Mb			Deletion: HEPACAM, KCNJ1, FLI1, JAM3, KIRREL3			
20	23	10p15.3p14 (Deletion)	10.1 Mb	*de novo*	DECIPHER 390248; DECIPHER 286091	Deletion: ZMYND11, GATA3, KLF6, WDR37	Dextrocardia, VSD	Bilateral choroid cysts	Termination
		16p13.3 (Duplication)	1.0 Mb	—	—	Duplication: NPRL3, CCDC78			
21	31	Xq27.2q28 (Duplication)	13.1 Mb	*de novo*	Xq27.3-q28 duplication syndrome	Duplication: FMR1, MECP2, NSDHL	RVE,TR, Coarctation of aorta	—	Termination
22	20	1p36.33p36.22 (Deletion)	10.9 Mb	*de novo*	1p36 deletion syndrome	Deletion: PRDM16	TOF	Hydrocephalus	Termination
23	26	5p15.33p15.31 (Deletion)	7.73 Mb	*de novo*	Cri-du-chat syndrome	Deletion: TRIP13, CTNND2, TERT, TRIO, SDHA	TOF	None	Termination
24	25	16p13.11 (Deletion)	1.22 Mb	*de novo*	16p13.11 microdeletion syndrome	Deletion: MYH11	VSD	None	Termination
25	24	17p13.3 (Deletion)	2.55 Mb	*de novo*	Miller-Dieker syndrome	Deletion: YWHAE, NXN, PAFAH1B1, PRPF8	DORV	None	Termination

CAVSD, complete atrioventricular septal defect (Single ventricle, common atrioventricular valve); DORV, double outlet right ventricle; PLSVC, persistent left superior vena cava; PTA, persistent truncus arteriosus, RAA, right aortic arch; RVE, right ventricular enlargement; TOF: tetralogy of fallot; TR, tricuspid regurgitation; VSD, ventricular septal defect.

The detection rate of pathogenic CNV in the isolated cardiac structural abnormality group was 6.6% (5/76), and in the group with associated extracardiac structural abnormalities, it was 12.79% (4/29). However, the difference between the two groups was not statistically significant (*P* > 0.05). Among the 16 fetuses with abnormal chromosomal karyotypes, CMA testing was conducted for 9 cases. In addition to confirming the specific locations of chromosomal variations detected by karyotype analysis, 4 fetuses (cases 6, 7, 11, 12) were also found to have additional microdeletions/microduplications not detected by karyotype analysis. Among them, case 12 was found to have a known pathogenic 1q43-q44 deletion syndrome.

### 3.4 Follow-up information

Pregnancy outcomes were observed and recorded for 105 CHD fetuses. Among them, 61.0% (64/105) were delivered, 36.2% (38/105) were terminated, and 2.8% (3/105) were lost to follow-up. The delivery rate in the isolated cardiac structural abnormality group (71.1%, 54/76) was higher than that in the group with associated extracardiac structural abnormalities (34.5%, 10/29), with a statistically significant difference between the two groups (*P* < 0.05). Among the 16 fetuses with abnormal chromosomal karyotypes, 87.5% (14/16) were terminated. Among the 89 fetuses with normal chromosomal karyotypes, 88.9% (8/9) of those with pathogenic CNV, 33.3% (3/9) of those with clinically unclear CNV, 20% (1/5) of those with benign CNV, and 18.1% (12/66) of those with normal CMA results were terminated. There was no statistically significant difference in termination rates among the various types of CNV (*P* > 0.05).

In this study, pregnancy outcomes and developmental follow-up were recorded for 64 delivered fetuses. Among them, 38.5% (10/26) of isolated ventricular septal defects (VSD) spontaneously closed within the first year of life, and three non-VSD cases also spontaneously resolved within the same period. Most infants showed no significant developmental delays; however, six cases (9.4%) exhibited varying degrees of intellectual or physical developmental delays. Three cases had mild intellectual delays with developmental quotients (DQ) between 70 and 85, and one case exhibited both physical growth delay and intellectual impairment, with a DQ score below 70, presenting symptoms such as poor head control and delayed motor milestones. Comparison between the two groups showed no statistically significant differences regarding spontaneous closure rates of isolated VSD or developmental outcomes (physical and intellectual) within the first year. Although genetic anomalies were detected through SNP array testing, the majority of these infants displayed favorable short-term outcomes, indicating the potential clinical benefit of early genetic evaluation. Long-term developmental outcomes beyond infancy require further evaluation (Table 4).

**Table 4:** Clinical data and genetic testing results of 6 cases of CHD patients with physical or intellectual developmental delay.

ID	delivery outcome	Physical development	Intellectual development	Cardiac & extra-cardiac conditions	Genetic testing results
17	Term birth at 36 weeks, Vaginal delivery, Birth weight: 2.8 kg	Delayed (Head circumference: Below; Weight: Below; Length: Average)	Normal (DQ: 90)	VSD	Normal karyotype, Pathogenic CNV (1.1Mb deletion in the 22q11.21q11.22 region)
26	Term birth at 38 weeks, Cesarean section, Birth weight: 3.2 kg	Normal (Head circumference: Average; Weight: Average; Length: Average)	Low (DQ: 78)	VSD, SUA	Normal karyotype, Normal CMA
27	Term birth at 39 weeks, Cesarean section, Birth weight: 2.2 kg	Delayed (Head circumference: Below; Weight: Below; Length: Below)	Below average (DQ: 65)	VSD (unclosed); LVE	Normal karyotype, VOUS CNV (3.1Mb deletion in the 12q14.3q15 region)
28	Term birth at 38 weeks, Cesarean section, Birth weight: 3.5 kg	Delayed (Head circumference: Average; Weight: Below; Length: Average)	Normal (DQ: 93)	PLSVC, Tricuspid regurgitation	Normal karyotype, Normal CMA
29	Term birth at 37 weeks, Vaginal delivery, Birth weight: 3.3 kg	Delayed (Head circumference: Average; Weight: Below; Length: Average)	Low (DQ: 82)	Atrial septal defect	Normal karyotype, Normal CMA
30	Term birth at 39 weeks, Cesarean section, Birth weight: 3.9 kg	Normal (Head circumference: Average; Weight: Above average; Length: Above average)	Low (DQ: 74)	Transposition of the great arteries (surgically corrected at 1 month)	Normal karyotype, Normal CMA

LVE, lateral ventricular enlargement; PLSVC, persistent left superior vena cava; SUA, single umbilical artery, VSD, ventricular septal defect.

## 4 Discussion

In this study, a total of 105 fetuses diagnosed with CHD through fetal echocardiography underwent karyotype analysis, revealing an overall chromosomal abnormality rate of 15.2%. Karyotype analysis identified chromosomal abnormalities in 16 CHD fetuses, with 13 cases of numerical abnormalities and 3 cases of structural abnormalities. Additionally, all 105 fetuses underwent SNP array testing, with a pathogenic CNV detection rate of 10.1%. Our data further demonstrate that, in addition to numerical and structural chromosomal abnormalities, chromosomal microduplication/microdeletion syndromes are significant contributors to CHD etiology. SNP arrays provide a valuable complement to routine chromosomal karyotype analysis, enhancing the detection of microdeletions and microduplications that may not be visible with traditional techniques. Our study demonstrates that SNP array analysis can detect submicroscopic chromosomal abnormalities that may not be identifiable through traditional karyotyping. This finding aligns with previous research indicating that SNP arrays enhance diagnostic yield in prenatal settings (22). Furthermore, through genetic tracing, we identified 3 cases of non-significant CNV. Follow-up of 64 delivered fetuses revealed only 6 cases with mild intellectual impairment, indicating that our findings may help avoid unnecessary inductions.

Chromosomal aneuploidy was the first recognized genetic factor in humans to be associated with CHD (23), with a high probability of CHD occurrence in individuals with trisomy 21, reaching 40%-50% (24, 25). In this study, chromosomal abnormalities were detected in 16 cases, with a detection rate of 15.2%. Among these, chromosomal numerical abnormalities accounted for 13 cases, with trisomy 21 and trisomy 18 being the most common, not completely consistent with previous reports (18, 26). The detection rate of chromosomal abnormalities in the group with combined extracardiac anomalies was significantly higher than that in the isolated cardiac structural anomaly group (31.03% vs. 9.21%). consistent with previous reports (18, 26, 27). This may be attributed to chromosomal numerical abnormalities carrying a large number of gene CNV, which, in addition to affecting heart development, may also be related to developmental abnormalities of other extracardiac organs such as the brain, kidneys, and skeleton (28). Additionally, In case 12, chromosomal karyotype analysis revealed a structural abnormality in chromosome 1, but the specific segment could not be identified. Through SNP array analysis, the variant site was found to include the 1q43–q44 deletion syndrome region. Cases 5, 6, and 7 were chromosomal aneuploidies, and in three cases, additional microduplications or microdeletions that could not be identified by karyotype analysis were detected through SNP array, indicating that fetuses with chromosomal abnormalities may also carry pathogenic CNV. Therefore, SNP array testing of CHD fetuses with chromosomal abnormalities can more accurately identify the location and nature of variants and may also reveal additional chromosomal segment abnormalities.

Conventional karyotyping necessitates viable cells, leading to longer turnaround times, increased risk of culture artifacts, and limited resolution (<5–10 Mb). In contrast, With its short TAT and high resolution, SNP array allows for comprehensive analysis of CNV throughout the genome, making it an excellent choice for detecting chromosomal abnormalities in prenatal samples. Therefore, the American College of Obstetricians and Gynecologists and Society for Maternal-Fetal Medicine recommend prenatal chromosomal microarray analysis for patients whose fetuses exhibit one or more major structural abnormalities identified during ultrasonographic examination and who are undergoing invasive prenatal diagnosis (29). This study conducted SNP array testing on 89 fetuses with normal chromosomal karyotypes, with a pathogenic CNV detection rate of 10.1% (9/89), like other experiments, our study confirming the viewpoint that SNP array technology can increase the detection rate of genetic causes of CHD fetuses compared to traditional chromosomal karyotype analysis techniques (18–20). Chromosomal microduplication syndromes are important causes of CHD, related to the effect of CNV on gene dosage or gene function (30). By analyzing the genes contained in pathogenic CNV through database and literature searches and comprehensive analysis based on gene haploinsensitivity, gene localization in human and animal models, and literature-reported gene function, it helps to screen candidate genes for CHD. In this study, SNP array testing identified 7 cases of known microdeletion/microduplication syndromes. Case 17 had a fetal chromosomal deletion segment, including most of the distal deletion syndrome 22q11.2. It has been reported that approximately 40% of patients with distal 22q11.2 deletion syndrome have CHD (31, 32), with the most common being ventricular septal defects. Case 18 had a 3.1Mb deletion, including the LCR22 A-D region of the 22q11.2 deletion syndrome. The 22q11.2 deletion syndrome is currently the most closely related microdeletion syndrome to CHD in humans, with a CHD occurrence rate as high as 75%. It is currently believed that the TBX1 gene in the LCR22 A-B region and the Crkl gene in the LCR22 C-D region are the main candidate genes for CHD (33). Studies have shown that haploinsufficiency of the MAPK1 gene between the LCR22 D-E regions leads to cardiac abnormalities in patients with distal 22q11.2 deletion. Fetal ultrasound in this study suggested tetralogy of Fallot and single umbilical artery (34). further corroborating the clinical relevance of the identified genetic variant. These findings underscore the utility of SNP array testing in elucidating the genetic basis of complex congenital anomalies and guiding clinical decision-making. CMA testing revealed 1p36 microdeletion syndrome in case 22, which includes the PRDM16 and SKI genes related to cardiovascular development, but further research is needed to verify the relationship between these genes and CHD (35, 36). Case 24 had a deletion at 16p13.11, including the MYH11 gene. MYH11 mutation can lead to abnormal aortic development, and some scholars abroad consider it as a predictive factor for thoracic aortic aneurysm and bicuspid aortic valve occurrence (37). Case 21 had a deletion segment including the Xq27.3-q28 duplication syndrome region, with FMR1 being an important pathogenic gene in this region, expressed more in the brain, and being an important gene causing intellectual disabilities (38). However, its expression in the heart is less, and whether there are pathogenic genes related to CHD occurrence in this region requires further study. Yan Wang et al (26). found that CHD with additional structural anomalies had a higher detection rate of pathogenic CNV than isolated CHD fetuses. However, this study showed that the detection rate of pathogenic CNV associated with extracardiac anomalies (13.8%) was higher than that of isolated cardiac structural anomaly group (6.6%), but there was no significant difference in statistics. This may be related to sample size limitations and the presence of more VOUS in this study, as well as factors such as parental refusal to undergo SNP array testing, which affected the determination of pathogenicity. Testing parental samples can assist in determining the nature of CNV and reduce the detection rate of VOUS. However, due to different data interpretation standards among institutions, a unified interpretation standard has not yet been established domestically, and the interpretation of VOUS still faces significant challenges.

Furthermore, Fetuses with chromosomal abnormalities and pathogenic CNV have a higher likelihood of harboring extracardiac structural anomalies and neurological developmental disorders. The comprehensive follow-up assessments conducted in our study allowed us to elucidate several key findings regarding the clinical implications of CNV detected through SNP array. Notably, we observed that a subset of patients with CNV exhibited only mild intellectual developmental delays[3 cases had mild intellectual delays with developmental quotients (DQ) between 70 and 85, and 1 case showed both physical growth delays and intellectual impairment, with a DQ score below 70]. This observation may hold significant implications for clinical decision-making, particularly regarding the necessity of interventions such as pregnancy termination. Our results demonstrate that most pregnancies involving fetuses with pathogenic CNV were medically terminated, highlighting the substantial impact of genetic findings on clinical decision-making. SNP array analysis has higher resolution than conventional karyotyping, increasing the detection rate of genetic etiologies in CHD fetuses. However, it also leads to a higher rate of VOUS, in this study, the VOUS proportion was 8.5% (9/105), which is relatively high but consistent with previous studies employing similar SNP array platforms (19, 39). Using a high-resolution genome-wide SNP array can yield a high diagnostic rate and reveal additional diseases (40). The termination rate associated with pathogenic CNV was 88.9% (8/9), compared to 20% (1/5) for benign CNV, indicating greater confidence among pregnant women and their families in continuing pregnancies without pathogenic findings. Consistent with previous studies, isolated VSD represented the most common form of CHD, with 38.5% spontaneously closing within the first year, in line with previous reports documenting spontaneous closure rates ranging from 44% to 80% (41, 42). Fetuses with isolated cardiac anomalies generally had low rates of genetic abnormalities and favorable outcomes. At the one-year assessment of 64 CHD infants, only six exhibited varying degrees of physical or intellectual developmental delays, including one case (case 17) carrying pathogenic CNV (22q11.2 deletion syndrome), which showed a relatively better developmental outcome compared to previous studies (19). Comparison of spontaneous closure rates of isolated VSD and developmental delays between groups revealed no statistically significant differences, suggesting generally positive short-term outcomes regardless of the presence of extracardiac anomalies. This underscores the potential benefits of early diagnosis and tailored management strategies in optimizing outcomes for CHD patients.

While our study provides valuable insights into the application of SNP array in fetal genetic diagnosis of CHD, several limitations should be acknowledged. Firstly, the sample size in our study was relatively modest, which may have influenced the generalizability of our findings. Additionally, as with any retrospective study, there is a potential for selection bias, particularly in the choice of patients who underwent prenatal diagnosis and SNP array testing. Moreover, the interpretation of variants of uncertain significance (VOUS) remains a challenge, as standardized criteria for classification are still evolving. Lastly, the follow-up period of one year may not capture long-term outcomes or late-onset manifestations of CHD, warranting further longitudinal studies.

In conclusion, SNP array analysis significantly enhances the prenatal genetic evaluation of fetuses diagnosed with CHD by increasing the detection rate of genetic anomalies. This approach facilitates a more accurate identification of genetic etiologies, thus assisting clinicians in making informed decisions regarding pregnancy management. Our findings indicate that fetuses with isolated cardiac structural abnormalities generally exhibit favorable developmental outcomes, and the majority of pathogenic CNVs detected were associated with extracardiac anomalies or severe phenotypes, influencing pregnancy termination decisions. Moreover, recognizing that some genetic abnormalities identified by SNP array are associated with only mild or no developmental impairments enables clinicians to provide more cautious and personalized counseling, potentially reducing unnecessary pregnancy terminations and alleviating psychological distress for families. However, it is important to note that establishing a direct causal relationship between identified genetic anomalies and specific cardiac malformations remains challenging. Although our study provides valuable insights into short-term developmental outcomes, further prospective studies with larger sample sizes and extended follow-up periods beyond one year are necessary to fully clarify the long-term developmental implications of genetic findings in fetuses with CHD.

## Data Availability

The datasets presented in this study can be found in online repositories. The names of the repository/repositories and accession number(s) can be found in the article/Supplementary Material.
